# NRF2 in dermatological disorders: Pharmacological activation for protection against cutaneous photodamage and photodermatosis

**DOI:** 10.1016/j.freeradbiomed.2022.06.238

**Published:** 2022-08-01

**Authors:** Shirin Kahremany, Lukas Hofmann, Arie Gruzman, Albena T. Dinkova-Kostova, Guy Cohen

**Affiliations:** a Department of Chemistry, Faculty of Exact Sciences, Bar-Ilan University, Ramat-Gan, 5290002, Israel; b The Skin Research Institute, The Dead Sea and Arava Science Center, Masada, 86910, Israel; c Jacqui Wood Cancer Centre, Division of Cellular Medicine, School of Medicine, University of Dundee, Dundee, UK; d Department of Pharmacology and Molecular Sciences and Department of Medicine, Johns Hopkins University School of Medicine, Baltimore, USA; e Ben-Gurion University of the Negev, Eilat Campus, Eilat, 8855630, Israel

**Keywords:** NRF2, UVA, UVB, Ultraviolet, Photodermatosis, Photocarcinogens, Photodamage, Pharmacological intervention, Sulforaphane

## Abstract

The skin barrier and its endogenous protective mechanisms cope daily with exogenous stressors, of which ultraviolet radiation (UVR) poses an imminent danger. Although the skin is able to reduce the potential damage, there is a need for comprehensive strategies for protection. This is particularly important when developing pharmacological approaches to protect against photocarcinogenesis. Activation of NRF2 has the potential to provide comprehensive and long-lasting protection due to the upregulation of numerous cytoprotective downstream effector proteins that can counteract the damaging effects of UVR. This is also applicable to photodermatosis conditions that exacerbate the damage caused by UVR. This review describes the alterations caused by UVR in normal skin and photosensitive disorders, and provides evidence to support the development of NRF2 activators as pharmacological treatments. Key natural and synthetic activators with photoprotective properties are summarized. Lastly, the gap in knowledge in research associated with photodermatosis conditions is highlighted.

## Highlights

• The deleterious action of ultraviolet radiation is summarized.
• UV-induced damage is aggravated in light sensitive disorders.
• Pharmacological activation of NRF2 can prevent photodamage and photocarcinogenesis.
• The limitations of targeting NRF2 is presented.

## Graphical abstract

**Figure:**
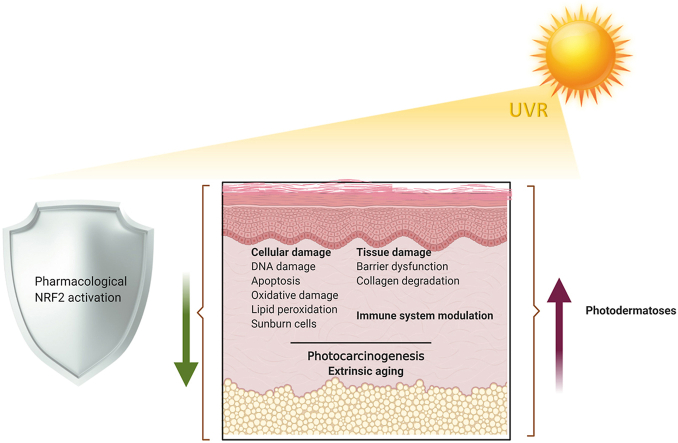

## Abbreviations list:

6–4 PP: Pyrimidine-(6–4)-pyrimidone
AP1: Activator protein-1
ARE: Antioxidant response element
caNRF2: constitutively active mutant NRF2
CGRP: Calcitonin gene-related peptides
CPDs: Cyclobutane pyrimidine dimers
cSCC: Cutaneous squamous cell carcinoma
ECM: Extracellular matrix
EM: Erythema
GST: Glutathione *S*-transferases
HO-1: Heme-oxygenase-1
IL: Interleukin
Keap1: Kelch-like ECH-associated protein 1
KO: knockout
MEF: Mouse embryonic fibroblast
mJ: Millijoule
MMP: Matrix metallo-proteinases
MoA: mechanism of action
NFκB: Nuclear factor Κb
NQO1: NAD (P)H: quinone oxidoreductase 1
NRF2: Nuclear factor erythroid 2-related factor 2
PPI: protein-protein interaction
RECQL: ATP-dependent DNA helicase Q-like
ROS: Reactive Oxygen Species
SPF: Sun protection factor
UVR: Ultraviolet radiation
UVR/A/B: Ultraviolet radiation, Type A and B
WT: Wild type
XP: Xeroderma pigmentosum

## 1 Introduction

Excessive or chronic exposure to the sunlight, and especially to ultraviolet radiation (UVR), adversely affects the skin and may cause erythema, barrier dysfunction, immune modulation, and premature extrinsic aging. In addition, prolonged exposure is linked to photocarcinogenesis and increased prevalence of skin cancer. In this review, we summarize the deleterious actions of UVR, and present evidence on the pivotal role of transcription factor nuclear factor erythroid 2-related factor 2 (NRF2) in the endogenous protection system of the skin. In addition, we highlight the beneficial protective role of this master regulator and summarize key pharmacological NRF2 activators that can prevent UVR-induced damage. We also explore the potential beneficial action of NRF2 in light-sensitive disorders as well as its involvement in certain cutaneous disorders. The limitations of targeting NRF2, mainly with respect to its potential tumorigenic action, is also presented.

## 2 The skin and ultraviolet radiation

**Fig. 1:**
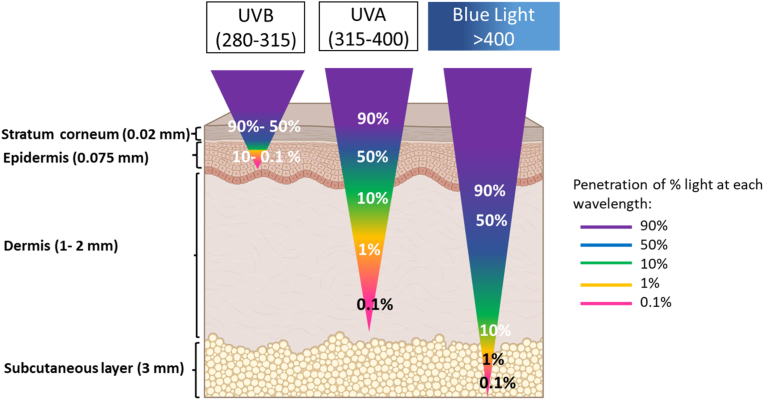
Skin penetration of UVB and UVA radiation, and blue light. The average thickness (forearm dorsal) of the skin layers is shown on the left which varies with age, sex, BMI, solar exposure, and anatomical site [15,16]. The wavelength-dependent penetration depth into the skin is represented in %, by color scale, and size [11,12]. All illustrations were created with Bioredner program. (For interpretation of the references to color in this figure legend, the reader is referred to the Web version of this article.)

The skin is the main barrier of the body, protecting it from physical, chemicals and biological threats while maintaining homeostasis. It is comprised of two main layers - the epidermis, and the dermis. Each maintains different parts such as hair roots, nails, nerve endings, sweat, and oil glands required to comprise the integumentary system [1]. The epidermal layer, and in particular the upper stratum corneum sublayer (Fig. 1), is responsible for the main physical barrier properties of the skin and typically blocks the entry of bacteria, chemical substances, and environmental stressors prior to systemic absorbance. The majority of the cells in this layer are keratinocytes that form desmosomes and tight junctions upon terminal differentiation, a structure that supports the skin barrier properties. The dermal layer beneath the epidermis consists of extracellular matrix required for the biophysical elastic properties of the skin, secreted mainly by the resident fibroblast cells [2].

Exposure to solar radiation can have short- and long-term manifestations on the skin, ranging from beneficial physiological processes, such as vitamin D synthesis, to detrimental effects, such as cutaneous photocarcinogenesis [[3], [4], [5]]. This solar energy or radiation is foremost optical radiation, and includes ultraviolet radiation (UVR), visible (light), and infrared radiation, albeit both shorter wavelength (ionizing microwaves) and longer wavelength (radiofrequency) radiation are present [6]. The wavelength of UVR is ranging from 100 to 400 nm, and is subdivided into UVA (315–400 nm), UVB (280–315 nm), and UVC (100–280 nm) [7]. The UV component of terrestrial radiation from the midday sun consists of about 95% UVA and 5% UVB, since UVC and most of UVB are filtered out by the stratospheric ozone [8,9], but may be modified according to altitude and ozone depletion. Several factors affect a person's UVR exposure flux and the concomitant effect; time of the day, season, geographic latitude, altitude, clouds and haze, ozone, air pollution, and ground reflection (for example snow reflects up to 80% of the UVR [10]). Knowing these factors allows to reduce the exposure to solar UVR to a certain degree. Nevertheless, complete avoidance is impossible. Therefore, protection means such as suitable clothing and high sun protection factor (SPF) containing formulations applied to the exposed areas of the skin provide additional means to reduce the detrimental effects of exposure to high or chronic solar UVR.

Both UVA and UVB radiation are capable of penetrating the human skin, albeit to different depths [11,12]. Penetration of UVR is wavelength- and location-dependent and may alter throughout the body, age, sex and BMI [12]. The higher energy, but low abundant UVB radiation, penetrates the stratum corneum and epidermis. Conversely, UVA reaches deeper into the dermal layer [11]. In general, approx. 50% of UVB and UVA radiation is blocked by the *stratum corneum* (SC) and the epidermis, respectively. Thus, only low UVB flux reaches the *stratum basale* in the epidermis, and only about 10% of the UVA radiation reaches the dermis (Fig. 1) [11]. Hence, the skin provides a highly efficient barrier against UVR. However, C–H or C–C bonds can already be broken with 75 kcal/mol (wavelength ≤381 nm) indicating that both UVA and UVB radiation are capable of causing severe damage, including DNA mutations [13,14]. Thus, current sunscreens intended to protect from UVR focus on both UVB and UVA radiation.

## 3 Ultraviolet radiation -induced photodamage

### 3.1 Barrier dysfunction

The skin and more specifically, the SC within the epidermal layer is the most exposed layer of the human body. 10.13039/100006132SC is the outermost layer of the epidermis and it is mainly surrounded by enriched lipid matrix which provides support to maintain proper 10.13039/100006132SC barrier integrity and function [17]. One of the important functions of the epidermis is to maintain homeostasis. For instance, regulation of water evaporation from the body to the atmosphere (*trans*-epidermal water loss (TEWL)) is imperative. In addition, the skin acts as the principal gatekeeper, reducing or blocking chemicals, pollutants, microbes and other environmental stressors systemic absorption [18]. This layer is also contributing to the protection from UVR. Although the effect of UVR on the epidermal barrier function is still incompletely understood, recent studies show that exposure to UVR, and mainly UVB radiation, affects epidermal morphology. This includes compensatory increase in the mean SC thickness, and disruption of the permeability barrier which causes morphological changes in SC lipids, and increased epidermal water loss [[19], [20], [21]]. The barrier properties are also tightly linked to keratinocyte differentiation, keratin synthesis and the generation of desmosomes, which are all regulated by cytokines and UV-induced inflammation in the skin (reviewed in Ref. [22]). In addition, it was shown that this exposure poses a threat to the skin by increasing biomechanical driving force for damage while decreasing the skin's natural ability to resist, compromising the critical barrier function of the skin [21]. Lipsky and German have recently determined the mechanical properties of isolated SC under UVR exposure and found that both UVA and UVB radiation cause mechanical and structural degradation [23]. Regardless of these harmful effects, UVB radiation was shown to have positive compensatory effects on the epidermal barrier when administered in low doses and over a relatively short period [24,25]. This therapeutic strategy is being used for the treatment of skin diseases with a disrupted epidermal barrier, such as atopic dermatitis, avoiding the possible side effects by applying only reduced intensity and exposure.

### 3.2 DNA damage, apoptosis, and photocarcinogenesis

Cutaneous DNA damage could occur through exposure to exogenous and/or endogenous carcinogens [26]. Both UVA and UVB radiation are capable of inducing DNA damage, though the former has a much broader range of damage. UVA radiation is considered to induce DNA damage primarily by production of highly reactive oxygen species which attack guanine bases [27], forming 7,8-dihydro-8-oxoguanine. UVB radiation is extensively absorbed by DNA, a process that produces DNA mutations such as cyclobutane pyrimidine dimers (CPDs) and pyrimidine-(6–4)-pyrimidone (6–4 PP) photoproducts [28]. Recent studies have shown formation of CPDs by UVA radiation as well as by the short wavelength region of visible light, but via a different route [29,30]. Due to the enormous carcinogenic potential of such DNA adducts, efficient DNA repair systems and cell cycle arrest mechanisms operate in the skin [31]. Caspase-3 activation and induction of apoptosis associated with UVR damage constitutes the last line of defense, eliminating damaged cells following p53 activation [32]. However, a tight regulation should be maintained to ensure that apoptosis rate will be sufficient to remove the damaged cells while still keeping the tissue regenerative and functional capacity.

The steadily increasing prevalence of melanoma, actinic keratosis (the most common pre-malignant manifestation of UVR-mediated skin damage, which may progress to squamous cell carcinoma), and non-melanoma skin cancer worldwide, and their association with UVR, illustrates the urgent need for development of novel and effective pharmacological interventions [33].

### 3.3 Erythema and immune modulation

The definition of erythema (EM) dates at least as far back as 1858, when the French neurologist Dr. Jean-Martin Charcot elaborated on solar EM [34]. By meticulous work and dissecting the spectrum of the sun, he demonstrated that the blue, violet and UV radiation is causing EM [35]. Nowadays, EM is understood as the result of hyperemia within the capillaries of the dermis and is manifested as redness of the skin. The increased blood flow can be caused by either inflamed or injured blood capillaries [36]. In general, it is classified as minor or major EM; while minor EM resolves itself within a few days, major EM requires professional treatment or hospital care. Currently, there are about ten known different types of EM (Table 1). The most investigated forms of EM are EM nodosum, followed by EM multiforme, EM chronicum migrans, and solar EM (according to PubMed research article count). However, solar erythema is arguably the most prominent form of EM, although it is under-represented and usually perceived as a cosmetic problem that typically resolves without medical intervention.

**Table 1:** Type of EM including most common causes, and corresponding publication count on PubMed high to low on the (April 20, 2022).

type of EM	most common causes	input search string	PubMed counts	selected references
**Erythema Nodosum**	*Streptococcal pharyngitis* infection	erythema and nodosum	4802	[[37], [38], [39]]
**Erythema Multiforme**	hypersensitivity to viruses or drugs	erythema and multiforme	4043	[36,40]
**Erythema Chronicum Migrans**	*Borrelia burgdor- feri* infection	erythema and chronicum migrans	1059	[41,42]
**Solar Erythema**	UVR	solar and erythema	778	[[43], [44], [45]]
**Palmar Erythema**	hereditary, hormones, or unknown	palmar and erythema	329	[46]
**Erythema Annulare Centrifugum**	dermatophytes and fungal infections	annulare centrifugum and erythema	299	[47,48]
**Erythema Ab Igne**	prolonged exposure of infrared radiation or other heat sources	Erythema and ab igne	224	[49,50]
**Fifth Disease (EM Infectiosum)**	viral infection of children	erythema and “fifth disease"	151	[51]
**Erythema Marginatum**	acute rheumatic fever	marginatum and erythema	118	[52]
**Erythema Toxicum**	unknown, mainly in newborns	erythema and toxicum	101	[53,54]

EM is a result of a complex overreaction of the immune system [39,55]. Because the underlying mechanisms are multifactorial and diverse, or not fully elucidated, current treatments are limited to nonsteroidal anti-inflammatory drugs for mild cases, or topical corticosteroids for severe cases [56].

Solar EM or sunburn is the major effect of extended and unprotected exposure to UVR [44,57]. In general, prolonged exposure to UVB radiation causes characteristic sunburn damage and represents a risk factor for carcinogenesis, compared to UVA radiation, which is mildly carcinogenic and mainly causes wrinkling and ageing of the skin [44,45,58,59]. After exposure to UVR, prostaglandins and reactive oxygen species are formed. Histamine, interleukins (IL-1, IL-6, IL-8), calcitonin gene-related peptides (CGRP), and TNFα are elevated eventually resulting in solar erythema accompanied by increased sensitivity beyond the affected area, due to mediator diffusion [[60], [61], [62]]. Interestingly, it was found that CGRP, Substance P, nitric oxide, and α-melanocyte-stimulating hormone build up an interconnected pathway modulating the inflammatory response to UVR in solar erythema [63]. Moreover, it was shown that following UVB radiation exposure, IL-1, IL-6, cyclooxygenase-2 (arachidonic acid metabolic enzyme), and CXCL5 (C-X-C motif chemokine 5) were upregulated, aside from many other chemokines responsible for the hypersensitivity and subsequent recruitment of macrophages and neutrophils [64,65]. The resulting immune response depends on the intensity, duration of UVR exposure (UV radiation flux) and skin type [57,66]. Conversely to the apparent upregulation of cytokines, it was shown that SE is accompanied by an endogenous negative feedback loop [63]. This immunosuppressive effect is caused by elevated IL-10 levels produced by keratinocytes and monocytes, which are also regulated by α-melanocyte-stimulating hormone [67,68]. Although the molecular mechanism underling UVR-induced immunosuppression is not fully elucidated, it is harnessed frequently as a treatment (phototherapy) in psoriasis and atopic dermatitis, when overstimulation of the immune response is present [[69], [70], [71], [72]]. Overall, solar UVR triggers production of various cytokines, causing inflammation, but simultaneously it regulates and balances this effect by immunosuppression.

### 3.4 Reactive oxygen species (ROS)

Reactive oxygen species (ROS) are highly unstable chemical species with unpaired electrons. Low levels of ROS can have hormetic or signaling effects, but high and uncontrolled levels impair the antioxidant defense system of the skin leading to oxidative stress and concomitant damage [60,[73], [74], [75], [76]]. The sources of ROS generation can be divided into two groups: exogenous sources which include UVR, food, and pollutants, and endogenous sources, such as diseases, mitochondrial dysfunction, and enzymatic activities of cytochrome P450 and NADPH oxidases [77,78]. ROS generation by UVR, and its effect on the skin, is a well-studied topic. Examples of UVR-derived ROS are oxygen radicals, including superoxide and hydroxyl radicals [79]. Moderate exposure of the skin to UVR is essential for the production of vitamin D which is involved in proliferation and differentiation of cells. However, as described above, higher exposure to UVR has deleterious effects on the skin, including through ROS generation, the concomitant generation of lipid peroxidation and macromolecule crosslinks, skin enzymes depletion, cytokines induction and formation of 8-oxo-2'-deoxyguanosine [[80], [81], [82]]. UVR-induced ROS generation can result in the manifestation of skin photoaging and even exacerbate the genotoxic action of UVB [83]. Mitochondrial DNA is particularly sensitive to UVR. As the main source of intracellular ROS is the respiratory chain, UVR-induced ROS generation can impair normal mitochondrial function [84]. Thus, a so-called ‘vicious cycle’ is generated leading to increased ROS generation and additional impairment of the mitochondria and the subsequent induction of apoptosis [85]. In addition, several studies show that pharmacological blockage of the respiratory complexes reduces and even normalized UVR-induced hydrogen peroxide accumulation in keratinocytes, emphasizing the importance of mitochondria in the deleterious action of UVR [86].

To overcome the damage caused by ROS, the skin has an extensive network of enzymatic and non-enzymatic defense systems. The enzymatic system includes antioxidants such as catalase, superoxide dismutase and glutathione peroxidase. The non-enzymatic system is comprised of various components of the skin with antioxidant activity such as vitamins E and C, glutathione, ubiquinol, and carotenoids [[87], [88], [89], [90]]. Thus, upon activation, the skin's endogenous defense can reduce or block ROS-dependent tissue and cellular damage.

### 3.5 ECM and photoaging

Fibroblasts, the main component of the dermis, are responsible for synthesis and secretion of extracellular matrix (ECM), including collagen, hyaluronic acid, and elastin. This compact network of ECM proteins and proteoglycans provides structural support to the skin and regulates a wide variety of signaling pathways, which control cell proliferation and differentiation (e.g., by binding to integrin receptors) and other processes critical to the skin function [91]. Skin aging that arises from exposure to external hazards such as UVR, is referred to as photoaging or extrinsic aging [92,93]. The molecular pathways underlying photoaging are complex and involve ECM proteins remodeling and alteration in the subcutaneous adipose tissue. One of the main players of photoaging are matrix metalloproteinases (MMPs). These proteolytic proteins are responsible for degradation of almost all ECM proteins [94]. To date, 24 different vertebrate MMPs have been identified in the human body. The members of this MMP super-family are secreted from the cell or anchored to the outer plasma membrane. MMPs are classified based on their domain organization into four groups: archetypal MMPs, matrilysins, gelatinases and furin-activatable MMPs [95]. The gene expression of MMPs is regulated by inflammatory cytokines, growth factors, glucocorticoids, or retinoids [96]. Some of the deleterious effects of ROS induced by UVR are also mediated by the activation of MMPs, in a MAPK/activator protein (AP)-1 and nuclear factor (NF)-κB dependent manner or by mutations in mitochondrial DNA [[97], [98], [99]]. In addition, ROS reduce TGFβ signaling and pro-collagen synthesis [100]. Altogether, the change of the ECM composition is tightly linked to both UVR-induced ROS and skin inflammation [101,102].

## 4 Photoprotective action of the NRF2 system in the skin

### 4.1 NRF2 and its regulation by Keap1

NRF2 (Nuclear factor erythroid 2-related factor 2) is a master regulator of the cellular stress response to environmental challenges [103]. As such, it is involved in the regulation of processes related to antioxidative defense, detoxification, inflammatory responses, proteasomal and autophagic degradation, and metabolism [104]. Consequently, its function is related to a vast number of chronic diseases such as cancer, neurodegenerative, cardiovascular, and metabolic diseases. In recent years, the role of NRF2 in skin cytoprotection has also become apparent. NRF2 belongs to the basic region leucine-zipper transcription factor family that binds to antioxidant response elements (AREs) in the DNA, thereby regulating the expression of over 200 genes that are involved in cellular defense against stress, such as GSTs (glutathione *S*-transferases), NQO1 (NAD(P)H: quinone oxidoreductase 1), and HO-1 (heme-oxygenase-1) [105,106]. Under normal conditions, NRF2 activity is suppressed by Kelch-like ECH-associated protein 1 (Keap1, a substrate adaptor protein for an E3 ubiquitin ligase complex) [107]. Keap1 interacts with two NRF2 sequences: a weak binding DLG motif and a strong-binding ETGE motif, and continuously targets NRF2 for ubiquitination and degradation in the proteasome [108].

The presence of electrophiles or oxidants activates NRF2 [109,110]. This activation occurs mostly through chemical modification of specific cysteine residues of Keap1, impairing its ability to target NRF2 for degradation. This causes NRF2 accumulation and nuclear translocation, and transcriptional induction of NRF2-target genes. In addition, activation of NRF2 can occur through phosphorylation, and several protein kinases have been shown to activate NRF2, such as PERK, AMPK, ERK [[111], [112], [113], [114]].

### 4.2 NRF2 in the skin

The importance of NRF2 in protecting the skin is evident from its expression profile in the layers of the skin. Based on the human protein atlas (https://www.proteinatlas.org), NRF2 is mostly expressed in the epidermal cells, mainly in the keratinocytes, melanocytes, and Langerhans and in the dermal layer. As the epidermis is the most exposed layer of the skin, NRF2 function is crucial for the integrity of the skin and protection against UVR. Consequently, it has been shown that the activity of NRF2 is increased during keratinocytes differentiation and in melanocytes, where high level of NRF2 synthesis was shown to be constantly maintained [115]. In the dermis, which mainly consists of fibroblasts, NRF2 is responsible of their proper functioning. Knockdown of NRF2 in murine fibroblasts significantly reduced their survival compared to cells derived from control mice [116]. In addition to epidermal and dermal cells, NRF2 is also expressed in hypodermal adipocytes. It was shown that NRF2 activity is essential for the regulation, formation and function of adipocytes, including lipid metabolism [117]. A critical role of NRF2 has also been found in the regulation of the pilosebaceous unit by the upregulation of the growth factor epigen, which was identified as a novel target of NRF2 in a study by Schäfer et al. [120]. However, it should be noted that chronic and constitutive hyperactivation of NRF2 may compromise its normal regulatory function leading to hyperproliferation, acanthosis and malformed hair pattens [118,119]. In addition, normal wound immune response and cutaneous redox balance is influenced by NRF2 [120]. Activation of NRF2 by cutaneous bacteria had also been suggested recently, providing evidence for a complex interplay with the skin microbiome [121]. In addition, it should be noted that the same harmful UVB radiation is also mandatory for vitamin D synthesis, that among other critical regulatory roles, activates NRF2 [122,123].

### 4.3 Evidence to suggest that endogenous NRF2 activation is required for a balanced UVR response

As mentioned above, the deleterious actions of UVR on the skin affect all its main functions. However, manifestation of the damage is restricted or blocked by endogenous repair mechanisms, and a tight balance between the need to counteract the damage and the normal functionality of the skin. As NRF2 modulates both inflammation and oxidative stress, it could be hypothesized that the damaging effects of UVR can be counteracted by increased endogenous NRF2 activity in skin resident cells. In addition, the normal compensatory acanthosis and hyperkeratosis seen in sun-exposed areas are mimicked in transgenic mice expressing a constitutively active NRF2 mutant (caNRF2) in keratinocytes [124,125].

**UVB radiation**: Overwhelming *in vivo* evidence from experiments performed mainly in NRF2-deficient mice, suggest that this pathway has an important role in photoprotection against UVB radiation. A study on NRF2 knockout mice (KO mice) showed that UVB radiation (200 mJ/cm^2^) induced a stronger and longer-lasting sunburn reaction with enhanced photosensitivity compared to wild-type (WT) controls. Additionally, histological images showed increased epidermal sunburn cells formation and presence of TUNEL-positive apoptotic cells after UVB radiation in NRF2 KO compared to WT mice [126]. Schäfer at el. established a novel strategy to study the function of NRF2 *in vivo*. They showed that increased NRF2-mediated gene expression in all layers of the epidermis strongly reduced the rate of UVB-induced apoptosis [127]. In a study by Hirota at el., it was demonstrated that UVB-irradiated NRF2 KO mice showed accelerated photoaging, such as coarse wrinkle formation, loss of skin flexibility and epidermal thickening [128]. Another study showed that UVB radiation (300 mJ/cm^2^) resulted in skin inflammation in both WT and NRF2 KO mice. However, the inflammation in WT mice returned to the basal level to a greater extent when compared to the KO mice [129]. In another study, the analysis of protein expression of numerous markers, such as macrophage inflammatory protein-2 (MIP-2), pro-matrix metalloproteinase-9 (MMP-9), and p53 in NRF2 KO mice after UVB radiation showed overexpression of these markers, indicating that the absence of NRF2 led to more sustained DNA damage [130].

Additional evidence for the involvement of NRF2 was provided by Schäfer et al. In their study, constitutively active mutant NRF2 (caNRF2, lacking the Keap1-binding site) was selectively expressed in keratinocyte cells. The transgenic animals exhibited enhanced endurance to UVB radiation (100 mJ/cm^2^), and epidermal apoptosis rate was reduced significantly. In addition, reduction of p53 expression was observed, indicative of reduced DNA and ROS damage [127]. In addition to the *in vivo* evidence, NRF2 had also been shown to be activated by UVR in keratinocytes, the main affected cell type by UVB radiation. For instance, Li et al. had shown that, in the well-established spontaneously immortalized HaCaT keratinocyte cell line, silencing NRF2 potentiated UVB-induced cell death [131]. However, others have shown reduced NRF2 response under UVR [132]. In addition, a complex interplay between keratinocytes and melanocytes, suggests a paracrine NRF2-dependent protection [133].

**UVA radiation**: Activation of NRF2 by UVA is still lacking sufficient evidence, and to date, no comprehensive *in vivo* studies have been performed. In studies on dermal fibroblasts, UVA radiation (10,000 mJ/cm^2^) resulted in accumulation of NRF2 in the nucleus after 2, 4 and 6 h. The researchers also showed that in contrast to the response to the UVA irradiation, nuclear accumulation of NRF2 is not induced by UVB irradiation [134]. The same observation of NRF2 activation was also seen by Zhong et al. [135]. A study on epidermal keratinocytes showed only slightly increased NRF2 expression and accumulation in the nucleus in HaCaT cells after radiation of 40,000 mJ/cm^2^ of UVA. Yet, Knockdown of NRF2 (siNRF2) strongly increased cell damage, as measured by membrane damage (LDH assay) and cell viability (MTT assay) following exposure to this dose of UVA irradiation [136]. By contrast, Ryšavá at el. concluded that keratinocytes show a less prominent response to UVA radiation for NRF2 translocation and NRF2-controlled proteins (HO-1, NQO1, GSR, GST, IL-6, and MMP-1, MMP-2), compared to primary human fibroblasts [137]. In a review by Ryšavá et al. the effect of UVR on the NRF2 signaling pathway in skin cells is well described [138].

The importance of NRF2 is further supported by experiments induced by solar-simulated radiation (UVA + UVB) in Keap1-knockdown SKH-1 hairless mice, which have ∼75% lower expression levels of Keap1 in their skin, and consequently increased levels of NRF2 and its downstream transcriptional targets; importantly, the level of NRF2 activation in these animals is comparable to that achieved by pharmacological inducers [14]. Compared to their wild-type counterparts, Keap1-knockdown mice have lower expression of the pro-inflammatory cytokines IL-6 and IL-1β and display reduced cutaneous erythema following acute exposure to solar-simulated UVR. Moreover, upon chronic exposure to solar-simulated UVR, tumor incidence, multiplicity and burden are substantially reduced in Keap1-knockdown mice in comparison with wild-type animals [14]. Conversely, the incidence, multiplicity and burden of cSCC that form in Keap1-knockdown mice that are also deficient for NRF2 are much greater than in their Keap1-knockdown/NRF2-proficient counterparts, establishing NRF2 activation as the protection mediator [139]. These observations are in agreement with studies in genetically modified mice expressing caNRF2 in specific cell types, which have demonstrated that by enhancing the production, recycling, and release of glutathione and cysteine by suprabasal keratinocytes, NRF2 activation exerts paracrine, glutathione and cysteine-dependent protection on the keratinocytes in the basal layer of the epidermis [127]. Lack of such protection in NRF2-deficiency may provide an explanation for the apparent paradox that whereas acute exposure to UVR causes greater epidermal necrosis, dermal edema, inflammatory cell infiltration, and oxidative DNA damage in the skin of NRF2-knockout in comparison to wild-type mice, there are no significant differences in cSCC formation between the two genotypes upon chronic exposure to UVR [140,141].

**Fig. 2:**
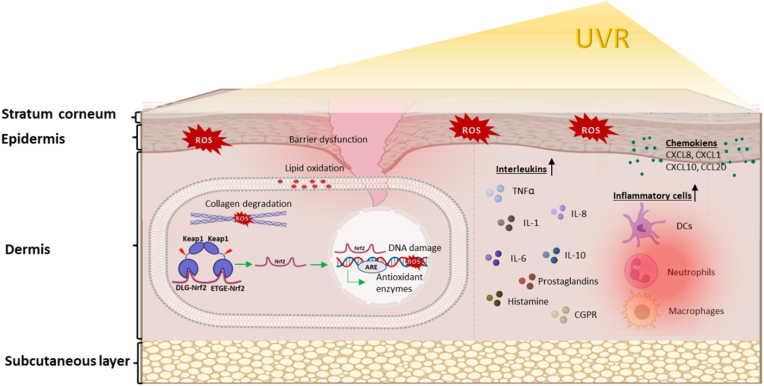
UVR-induced skin damage and NRF2. Exposure to UVR induces the formation of high levels of reactive oxygen species (ROS), which in turn can damage lipids, proteins, degrade collagen, and damage DNA (which is also damaged directly by UVB). ROS cause the activation of NRF2 and binding to its specific DNA sequences, antioxidant responsive elements (AREs), which however might be affected by UVR-induced mutations. Among the downstream targets of this transcription factor are genes encoding antioxidant and anti-inflammatory proteins.

Although induced by UVR (see Fig. 2), the extent of NRF2 activation is rather low in comparison to other stimuli [142]. The fact that NRF2 is activated after UVR exposure and the initial damage, may restrict its beneficial effect. As DNA adduct formation can precede NRF2 activation, transcriptional responses, such as ARE-dependent gene expression and protein synthesis, may be reduced or blocked. Thus, pharmacological pre-activation may be used to overcome this limitation. In addition, it should be noted that chronic photodamage with aging causes mottled skin pigmentation and solar lentigines and involves complex interactions between keratinocytes and melanocytes. Kerns et al. for example, have shown that the levels of NRF2 decrease in photodamaged skin, particularly in lentiginous skin of human subjects >45-years-old [143]. Notably, NRF2 activation is impaired with aging, with the loss of its electrophilic response and its targeted antioxidant cascades [144].

It should be noted that Nrf3, and not only NRF2, has been suggested to have a crucial role in a balanced response to UVR. Siegenthaler et al. explored the functionality of Nrf3 under UVR. In their study, keratinocytes of Nrf3-KO mice were less susceptible to UVR-induced apoptosis due to increase in cellular adhesion. This effect was independent of the classical NRF2 induction of antioxidant defense and the activation of its key target genes. In addition, a transient reduction in epidermal NRF2 expression was observed following physiologically relevant UVB radiation exposure (100 mJ/cm^2^) [145]. Thus, the tissue modulates its pro-apoptotic action to balance between the need to eliminate damaged cells and excessive induction of programed cell death. The overall role of Nrf3 in the skin is yet to be explored as well as its crosstalk with NRF2.

## 5 Pharmacological activation of NRF2 in the skin

The multiple damaging effects of exposure to UVR necessitate comprehensive strategies for protection [146]. This is particularly important when developing strategies for protection against cutaneous squamous cell carcinoma (cSCC), which is characterized by an extraordinarily high mutation burden [147] and altered epigenetic landscape characterized by hypermethylation [148]. Moreover, due to depletion of the stratospheric ozone, changes in lifestyle that lead to increased exposure to UVR and longer life expectancy, the prevalence of cSCC is increasing worldwide [149]. Therefore, in addition to improvements in the use of correctly applied high-quality sunscreen products [150], developing new strategies to prevent and treat cutaneous photodamage and photocarcinogenesis are urgently needed.

**Fig. 3:**
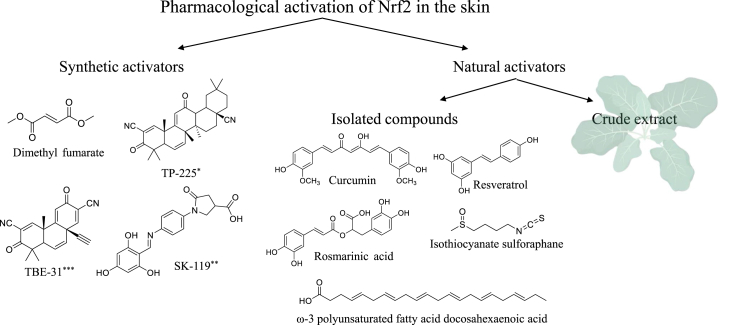
Representative NRF2 activators reviewed here. *Pentacyclic cyanoenone 2-cyano-3,12-dioxooleana-1,9(11)-dien-28-onitrile. **(E)-5-oxo-1-(4-((2,4,6-trihydroxybenzylidene)amino)phenyl)pyrrolidine-3-carboxylic acid. ***Acetylenic tricyclic bis(cyanoenone) (±)-(4bS,8aR,10aS)-10a-ethynyl-4b,8,8-trimethyl-3,7-dioxo-3.4b,7,8,8a,9,10, 10a-octahydrophenanthrene-2,6-dicarbonitrile.

NRF2 activation can be achieved using natural compounds or synthetic molecules (Fig. 3). Both of these categories include compounds which can be classified as electrophiles or protein-protein interaction (PPI) inhibitors [151]. The mechanism of action (MoA) of electrophilic compounds is by covalent interaction with cysteine residues of Keap1. This modification causes a conformational change in Keap1 and impairs its ability to target NRF2 for ubiquitination and degradation. As a result, the newly-synthesized NRF2 accumulates, translocate to the nucleus, and initiates target gene transcription. On the other hand, the MoA of PPI inhibitors is by interacting with the NRF2-binding site of Keap1 and disrupting the complex formation. All NRF2 activators are in fact “Keap1 inhibitors” since their mechanism of action involves engagement with Keap1 [152]. These compounds belong to distinct chemical classes, including cyanoenone triterpenoids, phenylenediamines, quinones, isothiocyanates and sulfoxythiocarbamates, thiocarbamates, dithiolethiones, polyenes, hydroperoxides, trivalent arsenicals, heavy metals and dimercaptans [[153], [154], [155]]. Some of these NRF2 activators have been shown to exert skin photoprotective effects, as summarized below.

### 5.1 Natural NRF2 activators

In recent years the use of natural NRF2 compounds for skin protection and dermatological disorders is becoming more prevalent [114]. These groups can be further subdivided into pure compounds or complex mixtures, such as plant extracts. The impact of the latter can be attributed to multiple compounds within it, and activated pathways, and therefore should be carefully examined. A number of natural NRF2 activators have been shown to protect against cutaneous photodamage and photocarcinogenesis. One example is the isothiocyanate sulforaphane, a phytochemical that was isolated from extracts of broccoli (*Brassica oleracea*) as the principal inducer of the classical NRF2-target enzyme NQO1 [156]. Sulforaphane is a hydrolytic product of the *S*-β-thioglucoside *N*-hydroxysulfate (glucosinolate) glucoraphanin [157]. Sulforaphane is produced upon plant tissue injury when a β-thioglucosidase enzyme known as myrosinase comes into contact with its substrate, glucoraphanin. It was subsequently shown that 3-day-old broccoli sprouts have much higher content of glucoraphanin than the mature plant [158]. Since then, pure glucoraphanin, sulforaphane, as well as highly standardized glucoraphanin- or sulforaphane-rich broccoli extracts have been used in numerous preclinical studies and clinical trials, including studies addressing their protective effects against cutaneous photodamage and photocarcinogenesis [159]. Thus, topical application to the skin of SKH-1 hairless mice of either pure sulforaphane or standardized broccoli sprout extracts containing an equivalent amount of sulforaphane, had quantitatively equivalent effects on the induction of NQO1 and on the inhibition of cutaneous edema and inflammation following exposure to UVR [160]. Moreover, when broccoli sprout extracts delivering ∼100 nmol/cm^2^ sulforaphane were applied daily to the skin of SKH-1 hairless mice that had been rendered high-risk for the development of cSCC by prior chronic exposure to UVR, ∼50% reduction in tumor incidence, multiplicity, and volume was observed, correlating with induction of cytoprotective responses in the skin of the animals [161]. Oral administration of glucoraphanin in this animal model was also protective: compared to control mice, tumor incidence, multiplicity, and volume were reduced by 25, 47, and 70%, respectively, in the animals that received the protective agent (10 micromol of glucoraphanin per day) in their diet [162].

Curcumin is a yellow pigment from turmeric (Curcuma longa) had been repeatedly suggested as a potent natural treatment in various diseases. Both topical and oral curcumin have been shown to reduce cancer formation following repeated UVB irradiation in SKH-1 hairless mice [163]. Li et al. had later found other photoprotective actions of curcumin, including reduced lipid peroxidation, lower immune cell infiltration and collagen remodeling. These protective actions were linked to NRF2 activation [164]. In addition, the study also demonstrates the protective effect in HaCaT keratinocyte cells as well as the reduction of DNA fragmentation induced by UVB radiation. Using shRNA directed to NRF2, a recent study by Deng et al. confirmed these results and provided additional evidence that similar protection was achieved by curcumin-induced NRF2 activation upon UVA radiation exposure. SOD, catalase, and HO-1 were all induced by curcumin, supporting the link between NRF2 activation and the resulting protection by curcumin treatment [165]. As curcumin has several pharmacokinetic drawbacks, microemulsion delivery system had been assessed to sustain optimal topical activity [166]. Of note, curcumin had been classified as pan-assay interference compound, affecting numerus biological pathways, and as a result may have low selectivity [167].

Topical administration of the flavanone naringenin, hesperidin methyl chalcone, or *trans*-chalcone protected mice from cutaneous photodamage [[168], [169], [170]]. Similar effects were observed with topical applications of the sesquiterpene zerumbone [171], a rosmarinic-acid containing extract from *Thymus vulgaris* [172], fisetin [173], shungite [174], grape stem extract [175], *Cordia verbenacea* extract [176], and even with supernatants of *Lactobacillus helveticus* NS8-fermented milk [177]. In each case, protection by these compounds correlated with NRF2 activation and enhanced transcription of genes encoding cytoprotective proteins, strongly suggesting that, in contrast to sunscreens, the observed protection is not due to the compounds themselves, but due to upregulation of the NRF2 transcriptional network.

The ω-3 polyunsaturated fatty acid docosahexaenoic acid has also been reported as a photoprotective agent in an NRF2-mediated induction of cytoprotective gene [178]. Topical application of this fatty acid reduced several key tissue morphological alterations induced by UVB radiation. HO-1 and NQO1 were induced by docosahexaenoic acid, correlating with lower levels of the lipid peroxidation product 4-HNE. Interestingly, UVA radiation increased the levels of two NRF2 inducers, 1-palmitoyl-2-(epoxyisoprostane-E2)-*sn*-glycero-3-phosphorylcholine and oxo-docosahexaenoic-acid, in docosahexaenoic acid supplemented cells, suggesting an autoregulatory action [179].

The photoprotection of resveratrol and its natural dimethoxy analog pterostilbene had also been explored *in vivo*; topical pre-treatments of SKH-1 hairless mice with both agents reduce EM and skin thickening upon exposure to UVB radiation. However, only pterostilbene dramatically attenuated the photocarcinogenesis process, and 90% of the pterostilbene-treated animals were tumor free in comparison to zero in the UVB radiation exposed control group. The authors excluded any direct sunscreen effect, as the compound had negligible SPF values. Supportive *in vitro* evidence showed a concentration-dependent activation of NRF2 [180].

Complex mixtures such as those of green tea polyphenols [181], also show promising photoprotection in an NRF2 dependent manner. In addition, either systemic (intraperitoneal) or topical administration of the apocarotenoid bixin was shown to protect against UVR-mediated cutaneous photodamage, as evidenced by reduction in oxidative DNA damage and inflammation in the epidermis of wild-type, but not of NRF2-KO mice, confirming the role of NRF2 in bixin-mediated cytoprotection [182]. Notably, topical bixin was also protective against PUVA (psoralen + UVA radiation)-induced hair graying [183].

Similar to mice, in humans, application of a single or multiple doses of broccoli sprout extract to small circular (1 cm in diameter) areas of non-sun-exposed skin, activates NRF2, as evidenced by an in increases the enzyme activity of NQO1 [184]. Importantly, this induction is long-lasting, and the NQO1 activity remains higher than that of the placebo-treated skin even when the skin biopsies were obtained 72 h after the application of a single dose of the extract. Furthermore, in a proof-of-principle study in six healthy human volunteers (three males and three females), the susceptibility to erythema (which was objectively determined 24 h after irradiation using reflectance spectroscopy) development following exposure to narrow-band (311 nm) UVR was decreased by ∼40% at those skin sites that had received 3 topical applications, 24 h apart, of sulforaphane-containing broccoli sprout extract in comparison with vehicle (80% acetone)-treated skin sites. In a larger double-blind placebo-controlled clinical trial in 24 healthy human subjects, the same dosing schedule led to reduction in erythema development caused by solar-simulated UVR at those skin areas that had been pre-treated with sulforaphane-containing broccoli sprout extracts in comparison with glucoraphanin-containing broccoli sprout extract-treated skin areas [141].

In addition to sulforaphane-rich broccoli extracts, pure sulforaphane was also effective at reducing the tumor multiplicity and burden in mice that had been co-treated with the compound and UVR [185]. During acute exposure to UVR, sulforaphane pre-treatment of wild-type mice restored the epidermal thickness to its basal levels, but this effect was blunted in NRF2-knockout animals, suggesting involvement of NRF2 in mediating the protective effect of sulforaphane [186]. Nonetheless, it is noteworthy that, in addition to activating NRF2, sulforaphane inhibits the transcription factor activator protein-1 (AP1) in the skin of mice exposed to UVR [185] and is also an inhibitor of transcription factor nuclear factor κB (NFκB) [187]. Considering that both AP1 and NFκB are key mediators of UVR-induced cSCC formation [188], it is likely that both activation of NRF2 and inhibition of AP1 and NFκB play roles in the protective effect of sulforaphane in this model.

Small-molecule NRF2 activators have also shown protective effects in animal models of cutaneous photoaging. Thus, it was shown that topical treatment of the dorsal skin of mice with the flavonoid galangin promoted the nuclear translocation of NRF2 and reduced the epidermal hyperplasia and senescence following exposure to UVB irradiation [189]. Protective effects against cutaneous photoaging due to chronic exposure to UVB were also observed with oral administration of resveratrol [190], sosihotang, a traditional Chinese remedy comprised of seven medicinal plants [191], or an extract of *Eisenia bicyclis*, a brown alga common to the middle Pacific coast around China, Korea, and Japan [192]. Interestingly, intraperitoneal administration of the lipoxin receptor/FPR2 agonist BML-111 reduced the UVB-induced cutaneous inflammation, decreased the epidermal thickness, and collagen degradation; this was accompanied by activation of NRF2 in the skin of the BML-111-treated mice [193]. Topically applied sulforaphane activated NRF2 in the mouse epidermis, decreased the UVA-mediated epidermal thickness, oxidative DNA damage, and the expression of matrix metalloproteinase-1 (MMP-1); this was accompanied by an increase in the levels of collagen, in agreement with the role that MMP-1 plays in collagen degradation [194].

Additionally, daily topical applications to photo-exposed skin of sulforaphane-rich broccoli sprout extracts for 7 days increased the NRF2 levels and reduced mottled hyperpigmentation and melanin deposition in the treated areas of the skin in 6 of 8 subjects; importantly, these effects were not observed in the two subjects, where NRF2 was not increased by the treatment, suggesting NRF2 dependence [143]. Using mouse models of UVB-induced hyperpigmentation, it was found that topical treatment of sulforaphane was effective in both preventing and treating UVB-induced hyperpigmentation in wild-type, but not NRF2-KO ear skin. Interestingly, the protective effect of sulforaphane was also lost in mice with keratinocyte-specific conditional IL-6Rα deficiency, implicating both NRF2 and IL-6Rα signaling in the sulforaphane-mediated protection [143]. These recent observations are in agreement with earlier reports of increased cutaneous levels of the lipid peroxidation product 4-hydroxy-2-nonenal and decreased levels of glutathione, and accelerated photoaging in UVR-exposed NRF2-knockout mice [195]. Together, these findings indicate that natural NRF2 activators can protect against UVB-induced cutaneous photoaging.

### 5.2 Synthetic NRF2 activators

**Fig. 4:**
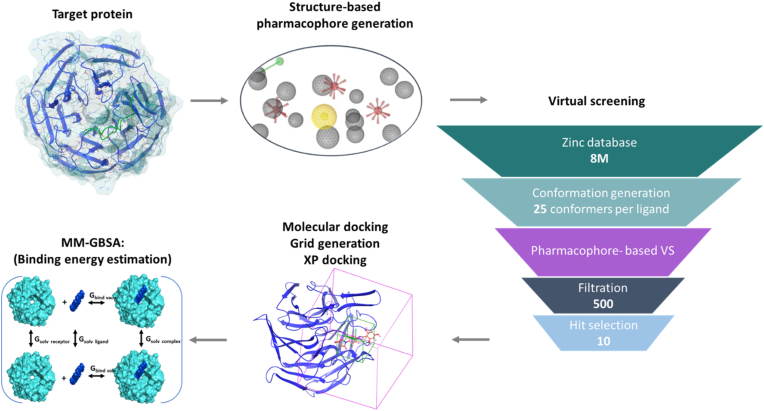
Overview of the *in silico* drug design workflow leading to the identification of the oxopyrrolidine-based class of NRF2 activators.

In recent years, synthetic NRF2 activators have been developed for skin protection [155,196]. The FDA-approved systemic drug, dimethyl fumarate, is used for the treatment of psoriasis and multiple sclerosis. The second most clinically advanced NRF2 activator for psoriasis treatment is under development by Annji Pharmaceutical and reached phase 2 clinical trials. This molecule was designed through extensive structure-activity relationship studies of curcumin to develop compounds having at least one (substituted phenyl)-propenal moiety [197,198]. To date, there is no approved NRF2 synthetic activator for skin protection against UVR. As stated here and in other studies, modulation of the NRF2 signaling pathway in the skin is of immense importance. The authors have recently used a computer-aided *in silico* approach to design and synthesize oxopyrrolidine-based class of NRF2 activators (Fig. 4) [199], and after *in vitro* examination, a lead compound SK-119 was generated [200,201]. The compound interacts with the arginine-rich area in the Kelch domain of Keap1 using the same binding mode as the NRF2–ETGE motif. SK-119 binds to the Kelch domain using two π–π stacking interactions between the compound and protein side chains (Tyr334 and Tyr572) and several H-bonds with the side chains (Arg415, Arg483, Ser508, Ser555, and Tyr334). The compound was designed to have good skin permeation properties to enhance its concentration in the active site (logP = 4.3, low molecular weight). For validation of its photoprotection properties, an *ex vivo* human skin organ culture was used [202,203]. The skin tissues are donated typically by women undergoing elective cosmetic surgery. After processing and removal of subdermal fat, the skin can be maintained in an air liquid interface for several weeks without reducing its viability or responsiveness [204,205]. Topically administrated SK-119 could activate NRF2 nuclear translocation. Importantly, the SK-119 treated skin was protected from UVB radiation-induced damage; UVR-induced ROS generation in the epidermal layer was completely blocked. In addition, apoptosis was attenuated in comparison to the UVB irradiated control skin and epidermal viability was enhanced. Similar to sulforaphane, the compound also reduced skin inflammation, measured by the secreted levels of IL-6 and IL-8. In addition, DNA damage was reduced and CPD levels were similar to the untreated control group [201]. Further animal and clinical trials are required to assess the full capacity of the compound. In addition, the impact of the newly developed compounds on UVR-induced vitamin D synthesis, and the overall regulatory processes described above of both NRF2 and ROS signaling cascades, should be extensively explored. Computer-based virtual screening coupled with chemical library screening was also performed by the group of Sabine Werner and found fourteen novel NRF2 activators [206]. Pre-treatment with the lead compounds reduced UVB radiation-induced cell death in primary human keratinocytes. Nitroxide derivatives that activate NRF2 have also been explored as topical photoprotectors [207]. Yet, their application is limited due to pharmacokinetics disadvantages that may be overcome by encapsulation [208]. Cinnamoyl-based Michael acceptor derivatives have also been tested for their ability to reduce photodamage. In their study Wondrak et al. showed that such compounds activate NRF2 and NRF2-target genes in both fibroblasts and keratinocytes *in vitro* and lower oxidative stress burden [209]. Similar to sulforaphane, other synthetic NRF2 activators have shown protective effects against skin photodamage and photocarcinogenesis in mice, including pure compounds, such as the pentacyclic cyanoenone 2-cyano-3,12-dioxooleana-1,9(11)-dien-28-onitrile (TP-225) [210] and the closely related tricyclic acetylenic cyanoenone TBE-31 [141].

Candidates for NRF2 activators were identified by *in silico* workflow. Structure-based pharmacophore model was generated from the crystal structure of the NRF2 ETGE peptide and the Kelch domain of Keap1 (PDB code: 2FLU) and prepared for virtual screening of ZINC database of eight million compounds. The compounds were filtered based on their fitting to the pharmacophore model and visual inspection. The top filtered compounds were then subjected to docking simulations followed by Prime MM-GBSA to estimate their binding energy to the receptor.

## 6 Photosensitivity and abnormal reaction to UVR – out of the frying pan and into the ire

For some, photodamage is aggravated by endogenous predisposition or exogenous factors. These photosensitive reactions may accelerate the deleterious action of UVR exposure by shifting the balance from accurate and beneficial repair responses to abnormal hyperactivation of the immune system or reduced repair capacity [211,212]. Generally, treatment of patients suffering from photodermatoses independent of the cause, are subjected to strict photoprotection, including limited exposure time, sun-protective clothing that reduce to a minimum the exposed areas [213].

### 6.1 Photosensitivity due to drugs and chemicals

Both topical and systemically applied drugs may react with the solar radiation and cause abnormal photosensitive reactions. The effects can be roughly divided into acute phototoxic or photoallergic reaction [214]. The former is more common and can be manifested within minutes to a few hours resulting in exaggerated sunburns, necrotic cellular damage and immune cell dermal infiltrations. On the other hand, photoallergic reaction symptoms are typically visible after longer duration and differ in both histological characteristics and clinical presentation of dermatitis after re-exposure to the drug. Both reactions are due to the inherent ability of the molecules to absorb the solar radiation, in most cases UVA radiation, and the generation of toxic (for instance, ROS such as superoxide or hydroxyl radicals) or immunogenic mediators (reviewed extensively in Ref. [215]). Thus, related problems can arise from both the amplification of oxidative stress and immune imbalance that are already induced by UVR. These drugs include commonly used agents, such as the cholesterol lowering statins (simvastatin and atorvastatin), thiazides and other anti-hypertensive drugs as well as some antibiotics (e.g., Tetracyclines) [216]. As some of the drugs are lifesaving or taken for a considerably long periods of time, reduced quality of life due to visual symptoms, as well restricted outdoor activities, is seen [217,218]. An extensive scientific effort is aimed to elucidate the cause (drug) responsible to the photosensitivity and alter the medication (see in Table 1, Erythema Multiforme), in order to increase patient compliance. Other photosensitizers can be both endogenous and exogenous. Photosensitivity originates from chemical molecules in cosmetic products or from pollution can be just as serous, yet can be limited or replaced relatively easily after identification of the photosensitizer [219,220].

The manifestations of drug- and chemical-induced photosensitivity are highly variable, but one common indicator is an exaggerated sunburn-like reaction in response to UVR exposure that normally would not cause damage [221]. Pruritus and pain are also common clinical symptoms in photosensitive conditions. These can be due to the severe blisters or accumulation of the transformed agent in the proximity of exposed nerve endings [222]. Other symptoms may include pseudoporpyria [223] (bulous dermatosis without porphyrin abnormalities) and drug-induced hyperpigmentation [224]. If a topical drug is used, the restricted burned area will be indicative to the underlying problem, however, identification of the cause can be challenging under a systemic drug regimen.

### 6.2 Photosensitivity due to immunological imbalance

The majority of photodermatosis conditions which are characterized by alteration in the immune response are idiopathic. Characteristically, the diseases are displayed on sun-exposed areas of scalp, face, neck, arms, hands, and back. Polymorphic light eruption, or “sun poisoning” is typically induced by UVB radiation; however, both UVA radiation and arterial light can induce a reaction. The underlying mechanism of this most common photosensitive disorder is still unclear, and it was found to be influenced by both genetic predisposition and environmental factors [225]. Symptoms, which include vesicles or plaques erythematous papules and pruritus, are apparent within hours of exposure [226]. First-line treatment, which in general is thought to all diseases in this section includes sun avoidance, broad SPF sunscreens and topical corticosteroids to reduce the inflammation. However, the user-defined low-dose sun exposure or narrowband UVB and UVA lamps had been shown to be effective, presumably due to the induction of endogenous tolerance and balanced immune response [227]. Chronic actinic dermatitis is another rather common immunologically mediated photodermatosis that affects the elderly population with high exposure to UVR [228]. The disease displays no genetic inheritance, affecting the population worldwide. It resembles allergic contact dermatitis with CD8^+^ cells infiltration. Treatment is still a challenge, but cyclosporine (calcineurin inhibitor) [229], azathioprine [230] and biological [231] drugs exist. Other rarer photodermatosis includes, actinic prurigo, hydroa vacciniforme and solar urticaria that also are caused by immune imbalance and are accompanied by pruritus.

### 6.3 Photosensitivity due to genetic deficiencies in DNA repair system

As written above, photocarcinogenesis is a result of the failure of the repair and checkpoint systems that deal with the majority of DNA mutations successfully. Thus, any reduced capacity of this system can lead to an increased prevalence of skin cancer. Indeed, one of the most severe photosensitive reactions to light is due to defects in the DNA repair mechanism. The best known genetic disease is xeroderma pigmentosum (XP). This autosomal recessive disease is characterized by the generation of numerous neoplasms (400-fold higher prevalence in comparison to the normal population) in sun-exposed areas, as well as ocular and cognitive abnormalities that affect both males and females [232]. The disease is diverse, composed of eight sub-groups according to the affected proteins that are responsible for all the major steps of global genomic repair, a subgroup of the nucleotide excision system. Mutation had been identified in proteins required for damage recognition (XPE and XPC), unwinding the damaged strand (XPB and XPD), stabilizing the site (XPA), removal of the damage (XPF and XPG) and in translesion DNA polymerase. To date, no treatment is available for this life-threatening disease, which results in premature death [233].

Bloom and Rothmund–Thomson syndromes are also photodermatosis conditions with genetic backgrounds. Both autosomal recessive diseases display with increase cancer formation that is not limited to the skin. Although rare, Bloom syndrome has a high prevalence among Ashkenazi Jewish but also ground in Japan and India and skin manifestation can be seen already in infants telangiectasias (“spider veins”) and erythema induced by sun exposure and later on in the increase prevalence of cancer. The syndrome is caused by mutation in the RECQL3 (ATP-dependent DNA helicase Q-like 3) DNA helicase, encoded by the BLM gene responsible to fix malfunctioning replication forks during DNA replication [234]. Rothmund–Thomson syndromes is due to a different helicase protein, RECQL4 (ATP-dependent DNA helicase Q4) with a pivotal role in genomic stability [235]. Both syndromes are managed by oncological guidelines, with a poor prognosis and premature death.

## 7 Pharmacological NRF2 activation in photodermatoses?

As light-sensitive disorders aggregate UVR-induced damage, it is clear that pharmacological intervention is required. This is true for the orphan genetic life-threatening diseases described above, but also for the less severe diseases to improve the patients' quality of life by enabling them at least some tolerance to the photosensitive cosmetic manifestations and the accompanied pruritus. Thus, a rationale for NRF2-based treatment directed to normalize the photodamage described above is highlighted here. Of note, similar conclusion was also suggested in light-sensitive retina disorders by Nakagami [236]. Not enough studies address these conditions and additional research is needed. To our knowledge, no attempt has been made to evaluate the impact of NRF2 activation to reduce photosensitivity disorders produced by immunological imbalance. Perhaps, the idiopathic nature of the disease, inappropriate drug screening models and low awareness are responsible to the lack of evidence. Yet, even in defined genetic diseases such as xeroderma pigmentosum, no in-depth studies have been conducted. However, one study has examined the potential of NRF2 activation to ameliorate photodermatoses; Kalra et al. explored the impact of TBE-31 (NRF2 activator) on photosensitization caused by azathioprine, an immunosuppressive drug widely used in inflammatory bowel disease and organ transplantations. Azathioprine increases the risk toward cSCC development due to its photosensitive 6-thioguanine metabolite [237]. In that study, mouse embryonic fibroblast (MEF) cells treated with TBE-31 were protected from UVA radiation-induced photosensitization. This was associated with the induction of NRF2 target protein NQO1. In addition, MEFs derived from Keap1-KO mice were highly protected despite having similar 6-thioguanine levels in their DNA, further demonstrating the protective effect of NRF2 activation. In addition, topical application of TBE-31 attenuated 6-thioguanine incorporation in the skin of azathioprine-treated animals. Notably, this strategy in drug-induced photosensitivity should be used with caution to ensure no systemic effects as they could potentially interfere with the therapeutic efficacy of the drugs.

## 8 Critical considerations in activating NRF2 as photodamage and photodermatosis treatment

As a key master regulator of detoxification enzyme as well as the cellular defense mechanism, upregulation of NRF2 may prevent photocarcinogenesis. However, one might ask what will happen if NRF2 is also activated in already established transformed cells or malignant tissues? It is possible that the activation in those cells may increase their adaptation to various stressors, leading to increased malignancy. Indeed, NRF2 mutations have been found in both melanoma and non-melanoma skin cancer, as well as its abnormal activation (typically associated with mutations in Keap1) [238,239]. In addition, in animals harboring a pro-oncogenic mutation in keratinocytes, constitutively activated NRF2 (caNRF2) enhanced tumor growth by modulation of the metabolic activity of the cells. This shift in metabolism as well as increase in key NRF2-target genes was also confirmed in human biopsies of actinic keratosis (the most common UVR-mediated pre-malignant manifestation) [240]. In a recent study, NRF2 activation enhanced melanoma malignancy by altering the balance between differentiation and proliferation as well as immune evasion both *in vitro* and *in vivo* in a cyclooxygenase-2-dependent manner [241]. Notably however, topical or oral administration of sulforaphane- or glucoraphanin-rich broccoli sprout extracts, respectively, to SKH-1 hairless mice after prior chronic exposure to UVR activated NRF2 and reduced the development of cutaneous squamous cell carcinoma [163,164], and co-treatment with pure sulforaphane during UVR exposure had a similar protective effect [186], showing that not all pharmacological NRF2 activators increase the risk for neoplasia, perhaps due to some of their other, NRF2-independent effects. Nonetheless, collectively these studies suggest that the timing of NRF2 activation should be critically considered, and its pharmacological activation primarily intended as a preventive strategy with close subsequent monitoring.

## 9 Concluding remarks

Compelling evidence suggests that preventive NRF2 activation may reduce cutaneous photodamage and photocarcinogenesis. The same approach could potentially be extended to photodermatosis, but knowledge in this area is insufficient. Perhaps a proof-of-concept clinical trial with sulforaphane may lead the way to this approach. In addition, if topical NRF2 activators could be used to complement existing SPF sunscreens in exposed areas of the skin, the application time prior of UVR exposure should be accurately considered to allow for induction of the downstream effector molecules. Such treatment would exceed the currently recommended 15-30-min application period prior to UVR exposure for commercial sunscreens. The generation of novel patentable NRF2 activators would facilitate the development of this protective strategy as drug companies would gain economic benefit over the non-patentable natural compounds. Beside pharmacological activation, skin-derived precursor cells, a population of dermal stem cells that participate in wound repair and cutaneous regeneration, are currently being explored as new photoprotective therapy. Several studies showed that injections of skin-derived precursor cells can reduce histopathological alteration caused by UVR as well as reduce apoptosis and oxidative stress in an NRF2/HO-1 dependent manner both in hairless mice and in 3D reconstructed skin equivalents [242,243].
